# Self-rated physical and mental health among older adults 80 years and older: cross-sectional results from a National community sample in Thailand

**DOI:** 10.1186/s12889-023-16237-y

**Published:** 2023-07-07

**Authors:** Supa Pengpid, Karl Peltzer

**Affiliations:** 1 Department of Health Education and Behavioral Sciences, Faculty of Public Health, Mahidol University, Bangkok, Thailand; 2 Department of Public Health, Sefako Makgatho Health Sciences University, Pretoria, South Africa; 3 Department of Healthcare Administration, College of Medical and Health Science, Asia University, Taichung, Taiwan; 4 Department of Psychology, University of the Free State, Bloemfontein, South Africa; 5 Department of Psychology, College of Medical and Health Science, Asia University, Taichung, Taiwan

**Keywords:** Older adults 80 years and older, Physical health, Mental health, Thailand

## Abstract

**Background:**

The aim of this study was to assess the self-reported physical health (SRPH) and self-reported mental health (SRMH) of older adults 80 years and older in Thailand.

**Methods:**

We analyze national cross-sectional data from the Health, Aging and Retirement in Thailand (HART) in 2015. Physical and mental health status was assessed by self-report.

**Results:**

The sample included 927 participants (excluding 101 proxy interviews), 80–117 years, median age 84 years [interquartile range (IQR) 81–86 years]. The median SRPH was 70.0 (IQR = 50.0–80.0), and median SRMH was 80.0 (IQR = 70.0 to 90.0). The prevalence of (good) SRPH was 53.3%, and the prevalence of (good) SRMH was 59.9%. In the final adjusted model, low or no income, living in the Northeastern, Northern and Southern region, daily activity limitations, moderate/severe pain, having one or two or more physical conditions, and low cognitive functioning were negatively associated, and higher physical activity was positively associated with good SRPH. No or low income, residing in the northern region of the country, daily activity limitations, low cognitive functioning, and probable depression were negatively associated with good SRMH, and physical activity was positively associated with good SRMH.

**Conclusion:**

SRPH and SRMH was relatively high rated among the oldest old in Thailand, and influenced by various social, economic, and health-related factors. Special attention should be given to those with no or low income, those living in the non-central regions and those having no or low formal social engagement. Health care and other services should improve physical activity, financial support, and physical and mental care management to promote physical and mental well-being of older adults 80 years and older in Thailand.

## Introduction

In Thailand the proportion of older adults (≥ 60 years) increased from 2 million in 1970 to 12 million in 2020, which is 18% of the total population [1]. In 2020, the life expectancy at 60 years for Thailand was 22.7 years; the older adults 80 years and older, 1.4 million (1.9% of the total population), will increase rapidly at the rate of 7% per year [2] to 3 million in 2039 [3]. In the older adults 80 years and older population, functional disability, multi-morbidity and health care needs and costs are likely much higher than in younger population groups [4]. Therefore, it of particular importance to assess the effectiveness of health care for the older adults 80 years and older [5]. Health care assessment commonly includes the evaluation of the health status, often assessed with a simple and generic instrument to evaluate the health status independent of specific diseases [5]. Self-reported physical health (SRPH) and self-reported mental health (SRMH) are important predictors of future morbidity and mortality and are important indicators for assessing health services and policy for the older adults 80 years and older [6–9]. Theories that may explain these findings may include that (1) Self-rated health (SRH) may represent ill-health that is difficult to assess using bio-medical means, (2) SRH may refer health risk behaviour and psychosocial context affecting health negatively, and (3) personality factors such as fatalism may influence health perceptions [10–12]. Based on previous studies [7, 13–17] on a conceptual model describing determinants of SRPH and SRMH, we propose socioeconomic status, sex, age, social capital, physical chronic diseases, body pain, functional status, mobility limitations, sensory deficits, cognitive impairment, depression, and health risk behaviours.

SRPH and SRMH (or health-related quality of life) may decrease with increasing age [18–20]. Among older adults 80 years and older in Sao Paulo, Brazil, 50.4% (0-100) had reported physical health summary and 43.4% (0-100) reported mental health summary [7]. Among older adults 80 years and older in Thailand in 2014, 34.4% rated their physical health as poor or very poor, the mean happiness score was 7.1 (0–10), and 22% had any daily activity limitations [21], and among older adults 80 years and older in Thailand in 2017 67% rated their physical health as good or very good [4].

Factors associated with better SRPH among older adults 80 years and older, include sociodemographic variables, including male sex [7, 22], older age [22], younger age (80–89 versus ≥ 90 years)] [7], higher subjective economic status, and region [22]. Health-related variables associated with higher SRPH are no, or lower activities of daily living [22], adequate access to medical service, no falls history in the past 12 month [7], good self-rated vision [7], physical mobility, physical activity [23], exercise [22], no pain, no anxiety/depression, no chronic illness [23], drinking alcohol [22] and adequate cognitive functioning [22]. Social and demographic factors associated with better SRMH (or subjective well-being) among older adults 80 years and older include age ≥ 100 (versus 80–89), female sex, higher education, ethnic majority group (versus minority), urban residence, number of children, financial independence, and access to adequate medical services [24]. Health-related variables associated with higher SRMH among older adults 80 years and older include leisure activities [24], good sleep quality [7], better self-rated health, lower functional disability score, no vision loss, no hearing loss [24], no medication use [7], exercising, past smoking (versus never smoking), current alcohol use (versus never drinking [24]), and adequate cognitive functioning [7].

Socioeconomic and regional differences may influence physical and mental health among older adults 80 years and older in Thailand. The gross regional product (GRP) was, for example, in the Northeast region 86,233 (Baht/year) GRP per capita, while in the Central region the GRP per capita was 265,663 (Baht/year) in 2020 [25]. The Human Development Index of 2019 in the four regions of Thailand was the highest in the central region 0.781 (Bangkok 0.841), followed by the southern region (0.766), the northern region (0.762), and the lowest in the northeastern region (0.758) [26]. Among older adults in Thailand, income distribution by region is highly unequal. Gini coefficient is highest in the northeastern region (0.64613), while lowest in the south (0.59334) [27]. Historically, although already the 1921 primary education Act established a three-year compulsory education within the primary level of seven years, which was enforced nationwide in 1935, enrolment was still low with an adult literacy of about 50% in the 1950s, this changed significantly after in 1951 the 1st National Scheme of Education (NSE) was adopted and a new National Education Plan was introduced in 1960 reaching adult literacy of more than 85.5% in the mid-1980s [28–30].

The Thailand Second National Plan for Older Adults (2002–2021) includes promoting “positive attitudes toward the elderly; strengthen health and economic services; and engage families, communities, and the private sector.” In terms of economics, establish an Older Adults Fund, pension fund for employees, and Old Age Allowance for the poor. Health services included “commitment to quality health and long-term care (LTC), with universal free health care, priority (‘green lane’) access to outpatient services for older adults, and establishment of dedicated older adult clinics in hospitals.” “The Plan introduced multidisciplinary home health-care teams; initiated the Community Volunteer Caregivers for the Older Adults project; established multipurpose Senior Citizen Centers, homes for older adults, and community learning centers; and initiated development of a community-based, integrated health care and social welfare services model for older adults” [31]. “Community hospitals provide treatment, while sub-district health promotion hospitals focus on preventative and restorative care. Care managers conduct a needs assessment based on the Barthel daily activity limitation index and develop a care plan for older people in their locality, and are then referred for LTC services, including home care” [4].

However, there is a lack of knowledge about the prevalence and associated factors of SRPH and SRMH among older adults 80 years and older in Thailand, which are needed to design health services and policies for the older adults 80 years and older [21]. To address this research gap, the aim of this study was to assess the SRPH and SRMH of older adults 80 years and older in Thailand in a cross-sectional study.

## Methods

### Sample and procedure

We analyze national cross-sectional data of older adults 80 years and older from the Health, Aging, and Retirement in Thailand (HART) study in 2015. In a three-stage (region, province, blocks or villages) stratified random sampling in each household, one person (≥ 45 years) was randomly selected, being the inclusion criterium. For frail respondents proxy interviews were administered [32, 33]. We restricted our analytical sample to those 80 years and older (N = 927), excluding 101 proxy interviews. The study received ethical approval from the “Ethics Committee in Human Research, National Institute of Development Administration – ECNIDA (ECNIDA 2020/00012)”, and participants provided written informed consent.

### Measures

#### Outcome variables

*Self-rated physical health status* was measured with the item, “In general, how would you rate your physical health status?” reported on a 0 (= very poor) to 100 (= excellent) visual analogue scale. Self-rated (good) physical health was defined as 70.0-100 (70.0 being the median).

*The self-rated mental health* s*tatus* was assessed with the question, “In general, how would you rate your mental health status?” reported on a 0 (= very poor) to 100 (= excellent) visual analogue scale. Self-rated (good) mental health was defined as 80.0-100 (80.0 being the median). The rating from 0 to 100 of the single item self-rated health status has been used and validated in previous studies [34–37] and found to have high predictive validity for mortality [8].

#### Social and demographic covariates

Variables included age, sex, education, marital status, region, religion, self or spouse owing a house, having a life insurance, and having private health insurance.

No or low income was calculated based on “annual income from employment, own business, agricultural /livestock /fishing business, short-term or contract work, financial support from family, renumeration/pension income from the government fund, occupational pension fund, private pension fund, social security / welfare income, income from government living allowance, veteran’s welfare benefit, other welfare assistance income, and income from other sources, and defined as the lowest quartile 0 to < 13,000 Thai Baht (average exchange rate in 2015: 1 US$=34.2 Baht)” [33].

*Low formal social engagement* (defined as absence of activity) was measured with 6 items on “religious, occupational, cultural organization, alumni or parent association or association of people from the same hometown, volunteer, and political organization” The question was, “In the past year, how often do you participate in these activities? Response options were “day, week, month, year, never.” [33, 38–40]. Responses were coded “1 = daily to at least once a month and 0 = once a year or never.” [40].

*Low informal social engagement* was asked with two items, (1) “In the past year, do you have any close friends or relatives who live nearby and have a close relationship with? (Please refer to the only person whom you meet most often)”, and (2) “If so, how often do you meet with them in person (number of times per day, week, month, year, other, never)?” “Low informal social engagement was defined as 1 = not having a close friend or relative or meeting a close friend less than once a month in the past year, and 0 = having a close friend or relative who lives nearby and has a close relationship with, and having met that person at least once in a month in the past year” [33].

#### Health-related covariates

*Daily activity limitations* were measured based on eating, bathing, dressing, and washing [41]. The response options ranged from 0= “able to do it all by myself” to 3 = “need help for all steps”. “Daily activity limitations were defined as any of the four items unable to do all by him or herself” (Cronbach’s α = 0.96).

*Body pain*. Past-month pain in 13 body parts (“the head, shoulders, arms, wrists, fingers, chest, abdomen, waist, hips, legs, knees, ankles, and toes” by asking the following question, “Did you feel any pain or ache in the following body parts in the last month?” The response options were none, mild, moderate, or severe. We defined moderate or severe pain in the past 4 weeks in one or more of the 13 body parts as “body pain” [42].

*Chronic physical conditions* (0, 1, 2 or more) were evaluated by self-reported health care provider diagnosed conditions, including “hypertension, diabetes, lung diseases, emphysema, cardiovascular diseases, heart disease, heart failure, rheumatism, arthritis, bone diseases, low bone density, osteoporosis, kidney diseases, cancer, and liver diseases” [43].

*Vision impairment* was determined from any of three questions, 1) “Have you been diagnosed by a doctor with visual impairment?’ (Yes/No), 2) Have you ever been diagnosed from a doctor with blind (1 eye), blind (2 eyes) (Yes/No), and 3) “How would you rate your current vision/eyesight?” from 0 = very poor to 100 = excellent, poor was classified as 0–50 and good as 60–100.

*Hearing impairment* was determined from any of three questions, 1) “Have you been diagnosed by a doctor with hearing impairment?’ (Yes/No), 2) Are you using a hearing device or aid? (Yes/No), and 3) “How would you rate your current hearing ability?” from 0 = very poor to 100 = excellent, poor was classified as 0–50 and good as 60–100.

*Cognitive functioning* was assessed with two tasks (i) word recall (immediate and delayed word recall tasks), and (ii) numeracy (serial-7), giving a total score of correct answers between 0 and 6. Low cognitive functioning was defined as 0–1 scores and high cognitive functioning 2–6 scores.

*Probable depression* (≥ 10 scores) was measured with the Center for Epidemiologic Studies Depression (CES-D-10) scale [44]. (Cronbach’s α = 0.80).

*The history of falls that affect physical health* was assessed in the last 2 years.

*Tobacco smoking* was assessed with the question, “Have you ever smoked cigarettes?” (response options: “1 = yes, and still smoke now, 2 = yes, but quit smoking, and 3 = never”).

*Alcohol use* was assessed with the question, “Have you ever drunk alcoholic beverages such as liquor, beer or wine?” (response options: 1 = yes, and still drinking now, 2 = yes, but do not drink now, and 3 = never).

*Physical activity* was sourced from questions on the frequency and duration of any type of exercise in the past week [45], and categorized as “none = inactivity, 1–149 min/week = low activity, and ≥ 150 min/week = high activity” [46].

#### Data analysis

Frequency and percentage distribution were calculated to describe the sample, and Pearson chi-square tests were used to test for differences in proportions. Logistic regression was used to estimate odds ratios and confidence intervals (95% CI) of SRPH and SRMH. Variables significant (*p* < 0.05) in univariable analysis were subsequently included in the multivariable model. Covariates were selected based on a previous literature review [7, 22–24]. All statistical analyses were performed with StataSE 15.0 (College Station, TX, USA). P < 0.05 was accepted as significant, and only complete cases were included in the analyses. Missing cases were < 2%, except for depressive symptoms (> 5%), therefore imputing the individual’s mean was applied to depressive symptoms. The variance inflation factor (VIF) was calculated to check for multicollinearity, and none was found between the study variables.

## Results

### Sample characteristics

The older adults 80 years and older sample included 927 participants (excluding 101 proxy interviews), 80–117 years, median age 84 years [interquartile range (IQR) 81–86 years]. The median SRPH was 70.0 (IQR = 50.0–80.0), and median SRMH was 80.0 (IQR = 70.0 to 90.0). The prevalence of (good) SRPH was 53.3%, and the prevalence of (good) SRMH was 59.9%. Univariable analysis showed that higher education, higher income, and region were associated with good SRPH and SRMH, and male sex was associated with good SRPH. Having a life insurance was negatively associated good SRPH. Low formal social engagement was negatively associated with good SRPH and SRMH (see Table 1).

**Table 1:** Sample characteristics by social and demographic factors, HART 2015

Variable	Sample	Self-rated physical health		Self-rated mental health	
	N (%)	N (%)	p-value	N (%)	p-value
All	927	484 (53.3)		543 (59.9)	
Age (in years) 80–89 90 or more	813 (87.7) 114 (12.3)	425 (53.4) 59 (52.7)	0.887	483 (60.7) 60 (54.5)	0.219
Sex Female Male	489 (52.8) 438 (47.2)	234 (48.9) 250 (58.3)	0.004	284 (59.4) 259 (50.5)	0.736
Married/cohabiting Divorced/separated/never married Widowed	311 (34.1) 48 (5.3) 553 (60.6)	154 (50.7) 25 (52.1) 301 (55.6)	0.368	182 (59.9) 26 (54.2) 326 (60.4)	0.703
Education No education Elementary >Elementary	152 (16.4) 719 (77.7) 54 (5.8)	61 (41.5) 390 (55.2) 33 (63.5)	0.003	72 (48.6) 438 (62.2) 32 (61.5)	0.009
Own house No Yes	101 (10.9) 826 (89.1)	47 (48.0) 437 (54.0)	0.261	50 (51.0) 493 (61.0)	0.057
Life insurance No Yes	788 (85.0) 139 (15.0)	423 (54.9) 61 (44.5)	0.025	464 (60.3) 79 (57.7)	0.556
Private health insurance No Yes	872 (95.5) 41 (4.5)	454 (53.1) 24 (58.5)	0.492	513 (60.1) 24 (58.5)	0.838
Religion Muslim and other Buddhist	55 (5.9) 871 (94.1)	26 (48.1) 458 (53.7)	0.428	31 (57.4) 512 (60.2)	0.688
Low formal social engagement No Yes	239 (25.8) 687 (78.8)	138 (59.2) 345 (51.2)	0.034	153 (65.9) 389 (57.8)	0.029
Low informal social engagement No Yes	786 (85.0) 139 (15.0)	415 (54.0) 68 (49.3)	0.309	457 (59.5) 85 (62.0)	0.577
Low or no income No Yes	587 (63.3) 340 (36.7)	132 (65.7) 352 (49.8)	< 0.001	142 (70.3) 401 (57.0)	< 0.001
Region Central North Northeast South	357 (38.5) 281 (30.3) 141 (15.2) 148 (16.0)	217 (61.0) 147 (53.3) 60 (43.2) 60 (43.8)	< 0.001	234 (66.3) 159 (57.2) 78 (56.9) 72 (52.2)	0.013

Daily activity limitations, moderate/severe pain, hearing impairment, low cognitive functioning, probable depression, and no physical activity were negatively associated with both good SRPH and SRMH. Higher number of physical conditions and visual impairment were negatively associated with SRPH (see Table 2).

**Table 2:** Sample characteristics by health-related factors, HART 2015

Variable	Sample	Self-rated physical health		Self-rated mental health	
	N (%)	N (%)	p-value	N (%)	p-value
Daily Activity Limitation No Yes	831 (90.9) 83 (9.1)	461 (56.6) 20 (24.1)	< 0.001	515 (63.4) 24 (28.9)	< 0.001
Moderate/severe pain No Yes	561 (60.5) 366 (39.5)	324 (58.9) 160 (44.7)	< 0.001	346 (63.0) 197 (55.2)	0.019
Physical chronic condition 0 1 2 or more	390 (42.1) 338 (36.5) 199 (21.5)	231 (60.9) 172 (51.5) 81 (41.5)	< 0.001	233 (61.5) 205 (61.4) 105 (54.4)	0.210
Visual impairment No Yes	571 (61.6) 356 (38.4)	350 (62.9) 134 (38.1)	< 0.001	358 (64.5) 185 (52.7)	< 0.001
Hearing impairment No Yes	689 (74.3) 238 (25.7)	393 (58.5) 91 (38.6)	< 0.001	435 (64.7) 108 (46.2)	< 0.001
Cognitive functioning High Low	705 (78.5) 193 (21.5)	412 (59.4) 64 (34.0)	< 0.001	461 (66.6) 69 (36.9)	< 0.001
Probable depression No Yes	789 (85.1) 138 (14.9)	434 (56.7) 50 (35.2)	< 0.001	484 (63.4) 59 (41.3)	< 0.001
Falls No Yes	837 (90.3) 90 (9.7)	444 (54.2) 40 (44.9)	0.098	488 (59.7) 55 (62.5)	0.605
Smoking tobacco Never Past Current	800 (86.3) 89 (9.6) 38 (4.1)	416 (53.3) 49 (55.1) 19 (50.0)	0.871	480 (61.5) 45 (51.1) 18 (47.4)	0.046
Alcohol use Never Past Current	836 (90.2) 68 (7.3) 23 (2.5)	437 (53.5) 31 (45.6) 16 (69.6)	0.130	498 (61.0) 30 (44.8) 15 (65.2)	0.029
Physical activity None 1-149 min/week ≥ 150 min/week	609 (65.7) 212 (22.9) 106 (11.4)	286 (47.8) 120 (58.8) 78 (73.6)	< 0.001	324 (54.6) 140 (67.6) 79 (74.5)	< 0.001

### Associations with self-rated physical health

In the final adjusted model, low or no income, living in the Northeastern, Northern and Southern region, daily activity limitations, moderate/severe pain, having one or two or more physical conditions, and low cognitive functioning were negatively associated, and higher physical activity was positively associated with good SRPH (see Table 3).

**Table 3:** Adjusted logistic regression with self-rated physical health, HART 2015

Variable	AOR (95% CI)	p-value
**Social and demographic factors**		
Sex Female Male	1 (Reference) 1.33 (0.97 to 1.81)	0.073
Education No education Elementary >Elementary	1 (Reference) 1.27 (0.82 to 1.97) 1.14 (0.53 to 2.89)	0.290 0.735
Life insurance	0.63 (0.41 to 0.97)	0.038
Low formal social engagement	0.81 (0.55 to 1.17)	0.259
Low or no income	0.63 (0.45 to 0.88)	0.006
Region Central North Northeast South	1 (Reference) 0.60 (0.40 to 0.89) 0.40 (0.24 to 0.65) 0.54 (0.31 to 0.91)	0.011 < 0.001 0.022
**Health-related factors**		
Daily Activity Limitation	0.41 (0.23 to 0.74)	0.003
Moderate/severe pain	0.63 (0.46 to 0.86)	0.004
Physical chronic condition 0 1 2 or more	1 (Reference) 0.61 (0.43 to 0.87) 0.36 (0.24 to 0.55)	0.007 < 0.001
Visual impairment	0.41 (0.29 to 0.56)	< 0.001
Hearing impairment	0.77 (0.53 to 1.11)	0.162
Low cognitive functioning	0.51 (0.34 to 0.76)	0.005
Probable depression	0.69 (0.45 to 1.08)	0.109
Physical activity None 1-149 min/week ≥ 150 min/week	1 (Reference) 1.30 (0.90 to 1.89) 1.92 (1.13 to 3.27)	0.164 0.016

AOR = Adjusted Odds Ratio

### Associations with self-rated mental health

In the final adjusted model, no or low income, residing in the northern region of the country, daily activity limitations, low cognitive functioning, and probable depression were negatively associated with good SRMH, and physical activity was positively associated with good SRMH (see Table 4).

**Table 4:** Logistic regression with self-rated mental health, HART 2015

Variable	AOR (95% CI)	p-value
**Social and demographic factors**		
Education No education Elementary >Elementary	1 (Reference) 1.21 (0.79 to 1.86) 0.74 (0.35 to 1.54)	0.384 0.414
Low formal social engagement	0.67 (0.45 to 1.01)	0.056
Low or no income	0.58 (0.41 to 0.80)	< 0.001
Region Central North Northeast South	1 (Reference) 0.61 (0.42 to 0.89) 0.64 (0.40 to 1.02) 0.63 (0.36 to 1.08)	0.011 0.058 0.090
**Health-related factors**		
Daily Activity Limitation	0.46 (0.26 to 0.82)	< 0.001
Moderate/severe pain	0.76 (0.55 to 1.05)	0.092
Visual impairment	0.78 (0.56 to 1.08)	0.130
Hearing impairment	0.68 (0.47 to 0.97)	0.038
Low cognitive functioning	0.37 (0.25 to 0.54)	< 0.001
Probable depression	0.54 (0.34 to 0.85)	0.008
Smoking tobacco Never Past Current	1 (Reference) 0.71 (0.35 to 1.44) 0.74 (0.32 to 1.72)	0.341 0.481
Alcohol use Never Past Current	1 (Reference) 0.61 (0.27 to 1.34) 1.03 (0.34 to 3.12)	0.215 0.953
Physical activity None 1-149 min/week ≥ 150 min/week	1 (Reference) 1.38 (0.93 to 2.03) 1.91 (1.09 to 3.34)	0.107 0.024

AOR = Adjusted Odds Ratio

## Discussion

This study aimed for the first time to provide national data on SRPH and SRMH among older adults 80 years and older in Thailand. We found a high median of SRPH (70.0) and median of SRMH (80.0). The latter may be compared to the mean happiness score of 7.1 (0–10) among older adults 80 years and older in Thailand [21] and the former with 67% of very good or good rated physical health among older adults 80 years and older in Thailand [4]. SRPH and SRMH seem to be higher in Thailand than among older adults 80 years and older in Sao Paulo, Brazil (50.4% physical health summary and 43.4% mental health summary) [7]. It is possible that some of these differences may be related to different forms of SRPH and SRMH measurements (in Thailand, a single item measure, and in Brazil a multi-item measure).

Furthermore, we found that low or no income, living in the Northeastern, Northern and Southern region, daily activity limitations, moderate/severe pain, having one or two or more physical conditions, visual impairment and low cognitive functioning decreased the odds, and higher physical activity increased the odds of good SRPH. No or low income, residing in the northern region of the country, daily activity limitations, hearing impairment, low cognitive functioning, and probable depression decreased the odds of good SRMH, and physical activity increased the odds of good SRMH.

Consistent with previous studies [22], we found among older adults 80 years and older that lower economic status and not residing in the central region had poorer SRPH. Some studies found that male sex and age [7, 22] were associated with good SRPH, while this study showed this association with male sex but not with age in univariable analysis. The latter was also found in a study among older adults 80 years and older in Sweden [23]. Regarding health-related variables, we found in agreement with previous research [7, 22, 23] that daily activity limitations, physical comorbidity, including vision impairment, body pain, and low cognitive functioning decreased the odds of SRPH and physical activity increased the odds of SRPH. Vision difficulties may adversely affect activities of daily living and thus impact negatively on SRPH. Although a previous study [23] found an association between anxiety/depression and lower SRPH, we found this to be true for probable depression only in the univariate analysis. We did not find an association between smoking, alcohol use and SRPH, as this was found in some previous research [22].

Consistent with some previous findings [7, 24], this study showed that a lower prevalence of SRMH in older adults 80 years and older with lower economic status, residing in the northern region of the country, daily activity limitations, physical comorbidity, including hearing impairment, lower cognitive functioning, probable depression and lower physical activity. Low formal social engagement decreased the odds of SRMH in univariate analysis. In a study among older adults (≥ 80 years) social functioning was associated with greater positive affect or better SRMH [47]. Hearing difficulties can adversely affect interpersonal communication and may trigger symptoms of anxiety and fear, inability to hear and/or see, thus negatively impacting SRMH [25]. Contrary to some previous research [24], we did not find any significant differences in the prevalence of SRMH regarding age, sex, ethnicity or religion, smoking and alcohol use. Higher education was associated with better SRMH in China [24], while we only found this in univariable analysis. Lower socioeconomic status can be considered as a chronic stressor, as lower socioeconomic status may perpetrate various adverse social and environmental conditions [48]. Lower educational attainment likely affects health both through a higher-stress lifestyle and through material deprivation.” [49]. In terms of educational attainment in Thailand, there was low school enrollment in the 1960s [29], meaning that the older generation had less educational opportunities. In our study, 17.7% of the older adults 80 years and older had no formal education.

We found regional differences in the prevalence of SRPH and SRMH, with the northeast region, northern and southern regions scoring lower than the central region in both SRPH and SRMH. These differences seem to be reflected in regional socioeconomic inequities, with the northeastern region having the lowest GRP, lowest Human Development Index and highest Gini coefficient, and the central region having the highest GRP and highest Human Development Index [25–27], and the central region including Bangkok having a lower poverty rate than all other regions [4]. Implications of these findings are that health care planning for older adults 80 years and older should consider these regional differences. Lower cognitive functioning decreased both SRPH and SRMH. Cognitive loss may lead to a decrease in autonomy and social interaction, negatively affecting SRPH and SRMH [7]. Physical activity was found to positively affect both SRPH and SRMH, which may be linked to the release of emotion-related neurotransmitters [45], and should be promoted among older adults 80 years and older. The Thai government may want to increase its “pension coverage and benefits to address the income insecurity of older people. The universal allowance for older people should relate to the national subsistence level and the poverty line.” [50].

### Study limitations

The survey excluded institutionalised older adults 80 years and older. SRPH and SRMH were only assessed with single items. Cognition was only assessed with two components, and future studies should include multi-component cognitive measures. Depression was only assessed with a screening instrument. Nutrition may influence health problems among older adults 80 years and older, but this was not assessed in this study and should be part of future research.

## Conclusion

SRPH and SRMH was relatively high rated among the oldest old in Thailand, and influenced by various social, economic, and health-related factors. Special attention should be paid to those without or low income, those living in non-central regions, and those who have no or low formal social participation. Health care and other services should improve physical activity, financial support, and physical and mental care management to promote physical and mental well-being of the older adults 80 years and older in Thailand. For example, physical activity interventions could include ≥ 150 min/week moderate intensity activity such as brisk walking, ≥ 2 days/week muscle strengthen activities, and activities such as standing on one foot to improve balance, and in terms of mental health, interventions can include active screening for depression and home-based depression care management.

## Data Availability

Data is publicly available at Gateway to Global Ageing Data, Health, Aging, and Retirement in Thailand: https://g2aging.org/?section=study&studyid=44.
